# Candidate Genes Associated with Delayed Neuropsychomotor Development and Seizures in a Patient with Ring Chromosome 20

**DOI:** 10.1155/2020/5957415

**Published:** 2020-01-21

**Authors:** Thiago Corrêa, Amanda Cristina Venâncio, Marcial Francis Galera, Mariluce Riegel

**Affiliations:** ^1^Genetics Department, Post-Graduate Program in Genetics and Molecular Biology, Universidade Federal do Rio Grande do Sul (UFRGS), Porto Alegre, RS, Brazil; ^2^Post-Graduate Program in Health Sciences, Universidade Federal do Mato Grosso (UFMT), Cuiabá, MT, Brazil; ^3^Department of Pediatrics, Universidade Federal do Mato Grosso (UFMT), Cuiabá, MT, Brazil; ^4^Medical Genetics Service, Hospital de Clínicas, Porto Alegre, RS, Brazil

## Abstract

Ring chromosome 20 (r20) is characterized by intellectual impairment, behavioral disorders, and refractory epilepsy. We report a patient presenting nonmosaic ring chromosome 20 followed by duplication and deletion in 20q13.33 with seizures, delayed neuropsychomotor development and language, mild hypotonia, low weight gain, and cognitive deficit. Chromosomal microarray analysis (CMA) enabled us to restrict a chromosomal segment and thus integrate clinical and molecular data with systems biology. With this approach, we were able to identify candidate genes that may help to explain the consequences of deletions in 20q13.33. In our analysis, we observed five hubs (ARFGAP1, HELZ2, COL9A3, PTK6, and EEF1A2), seven bottlenecks (CHRNA4, ARFRP1, GID8, COL9A3, PTK6, ZBTB46, and SRMS), and two H-B nodes (PTK6 and COL9A3). The candidate genes may play an important role in the developmental delay and seizures observed in r20 patients. Gene ontology included microtubule-based movement, nucleosome assembly, DNA repair, and cholinergic synaptic transmission. Defects in these bioprocesses are associated with the development of neurological diseases, intellectual disability, neuropathies, and seizures. Therefore, in this study, we can explore molecular cytogenetic data, identify proteins through network analysis of protein-protein interactions, and identify new candidate genes associated with the main clinical findings in patients with 20q13.33 deletions.

## 1. Introduction

Ring chromosomes are rare structural rearrangements in humans, exhibiting an estimated frequency of 1 in 25,000 recognized conceptions [1] and approximately 1 in 30,000–60,000 births [2]. The ring formations are generally *de novo*, being only 1% inheritable [3–5]. Ring chromosome 20 (r20), which was first described in 1972 [6], is a syndrome characterized by refractory epilepsy, intellectual impairment, and behavioral disorders. Facial dysmorphism or other congenital malformations are rarely reported. r20 patients may present normal development until seizure onset, with the cognitive-behavioral decline being observed later, which suggests that the syndrome can be considered an epileptic encephalopathy [7, 8]. Most likely, r20 formation is the result of intrachromosomal fusions from the direct union of unstable telomeres or the occurrence of two breaks, one in each chromosomal arm, resolved by the junction of the telomere ends of both arms, short and long, forming a circular structure [9, 10]. In the latter case, deletions, duplications, and/or inversions usually occur at the chromosomal ends [11, 12]. The diagnosis of ring chromosome 20 syndrome requires identification of ring formation by conventional cytogenetic techniques with the complement of chromosomal microarray analysis to detect small losses and gains in genetic material.

There are fewer than 20 cases described in the literature of patients carrying subtelomeric deletions in 20q13.33 [13–18]. Common manifestations of these individuals include skeletal and growth abnormalities, behavioral problems, developmental delay, and seizures [19]. However, there are at least three factors that impair the clinical characterization of affected individuals and the identification of causal genes. First, there are notably few individuals molecularly characterized with high-resolution techniques. Second, there is no pattern regarding the presence of specific clinical manifestations in 20q13.33 deletions. Third, the significance of the ring morphology or chromosomal duplications in clinical abnormalities is unknown. These factors hinder efforts to explain the pathogenesis of 20q13.33 deletions and the probable molecular mechanisms involved in the phenotypic presentation of these individuals.

In this report, we describe an individual carrying a ring chromosome 20 with duplication and deletion in 20q13.33. The integration of cytogenetic, clinical, and protein-protein interactions data enabled us to identify genes that help to explain how the patient's phenotype is affected by the 20q13.33 deletion present on the ring chromosome.

## 2. Case Presentation

The proband is female, aged 2 years and 8 months, and is the only daughter of nonconsanguineous parents. The 35-year-old father and the 29-year-old mother reported gestation with high blood pressure and cesarean section with a gestational age of 41 weeks. The girl was born with a weight of 2860 g, length of 45 cm, and cephalic perimeter of 33 cm. The first convulsion of the child was manifested at 15 days of life as a generalized epileptic crisis, which was repeated 24 hours later. At 21 days, new seizures were characterized as focal seizures with secondary generalization and treatment with phenobarbital. The first neuropediatric assessment occurred at 4 months, showing delayed neuropsychomotor development, mild hypotonia, low weight gain, and cephalic perimeter of 36 cm (<−3 *Z* scores). The electroencephalogram, cranial resonance, and screening tests for inborn errors of metabolism were unchanged. The child was referred for genetic evaluation. At the age of examination, the patient showed some facial dysmorphic features as enlarged nasal dorsum, bulbous nasal tip, short *columella*, and long nasolabial filter. The treatment with phenobarbital was efficient with control of the epileptic seizures for approximately 1 year, when it presented decompensation of the convulsive conditions, making it necessary to change the anticonvulsant to oxcarbazepine and levetiracetam. The child started walking independently at 26 months, and at 2 years and 6 months of age, the child presented delayed motor development and language and high cognitive deficit.

**Figure 1:**
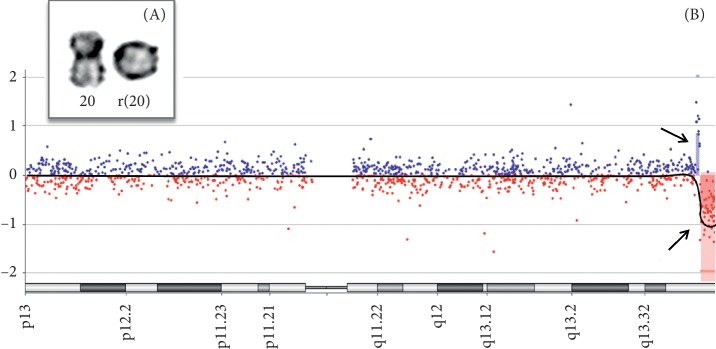
(A) Chromosome banding showing the chromosome 20 pair, with a normal chromosome and the other on the ring (20). (B) CMA profile of chromosome 20 showing the hybridization pattern. The genomic segment with a gain of ∼302,774 kb (61,142,577–61,445,350) is shown (vertical blue band). The arrow indicates the shift of the median ratio log^2^ to +1.0. The segment with loss of ∼1.4 Mb (61,472,348–62,872,839) is demonstrated (vertical red band). The arrow indicates the shift of the median ratio log^2^ to −1.0.

**Figure 2:**
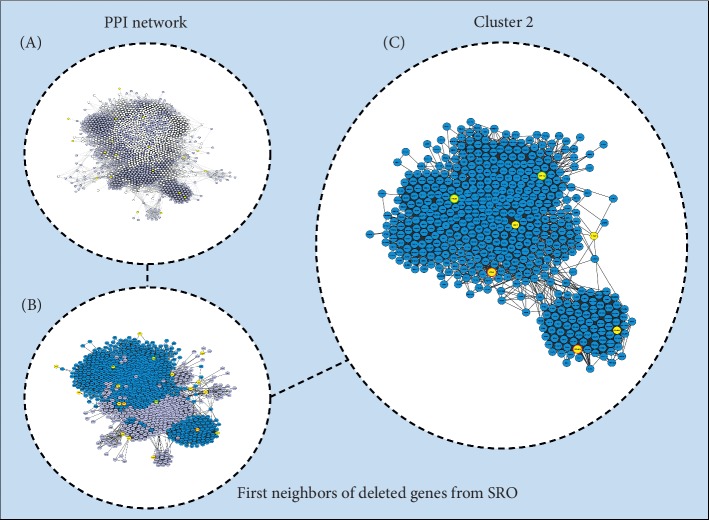
PPI network. A list of 40 genes and gene predictions was obtained from the human assembly of February 2009 (GRCh37/hg19) ([Supplementary-material supplementary-material-1]). Interaction data from STRING were used to construct networks using cytoscape software. (A) The primary network is composed of 1,572 nodes (proteins) and 54,925 edges (interactions). (B) The secondary network is composed of 794 nodes and 16,805 edges; yellow nodes are candidate proteins with data available in string (ZGPAT, HELZ2, EEF1A2, TNFRSF6B, ZBTB46, DIDO1, ARFGAP1, RTEL1, SRMS, GMEB2, YTHDF1, PTK6, GID8, BIRC7, COL9A3, COL20A1, KCNQ2, ARFRP1, and CHRNA4). (C) Cluster 2, with *C*_*i*_ = 40,863, is composed of 510 nodes and 10,388 edges. Nodes with green borders are considered hubs; orange are bottlenecks; and red are hub bottlenecks.

Karyotyping from the proband was performed on metaphase spreads prepared from peripheral blood samples. The chromosomal analysis was conducted after GTG banding at 550-band resolution, and at least 100 cells were analysed (Figure 1A). The karyotype showed results 46,XX,r(20). The parental karyotype was normal. At least 100 cells from each individual were analyzed. The DNA sample from the child was investigated using chromosomal microarray analysis (CMA) with a 60-mer oligonucleotide-based microarray with a theoretical resolution of 40 kb (8 × 60 K, Agilent Technologies Inc., Santa Clara, CA, USA). The images were analyzed using Cytogenomics v 2.0 and 2.7 with the statistical algorithm ADM-2 and a sensitivity threshold of 6.0 (Figure 1B). It is recommended to provide confirmation of CMA results with other methods such as FISH or real-time PCR, especially for the refinement of breakpoints of structural chromosomal abnormalities. The authors pursued to perform it; but unfortunately, there is no sample left of this patient anymore, and the family is not available to obtain a new sample. The protein-protein interaction (PPI) metasearch engine STRING 10.0 (http://string-db.org/) was used to create PPI networks based on 40 genes and gene predictions located in the deleted region from our patient (Figure 2A). The list of genes was obtained from CMA analyses and subsequent research into the human assembly of February 2009 (GRCh37/hg19) [20, 21]. The parameters used in STRING were (i) degree of confidence, 0.400, with 1.0 being the highest level of confidence; (ii) 500 proteins in the 1st and 2nd shell; and (iii) all prediction methods enabled, except for textmining and gene fusion. The final PPI network obtained through STRING was analyzed using Cytoscape 3.5 [22]. The MCODE tool was used to identify densely connected regions in the final Cytoscape network. The PPI modules generated by MCODE were further studied by focusing on major biology-associated processes using the Biological Network Gene Ontology (BiNGO) 3.0.3 Cytoscape plugin [23]. The degree of functional enrichment for a given cluster and category was quantitatively assessed (p value) using a hypergeometric distribution. Multiple test correction was also implemented by applying the false discovery rate (FDR) algorithm [24] at a significance level of *p* < 0.05. Finally, two major parameters of network centralities (node degree and betweenness) were used to identify hub-bottleneck (H-B) nodes from the PPI network using the Cytoscape plugin CentiScaPe 3.2.1 [25].

## 3. Discussion

The underlying biological mechanism in individuals with ring 20 has not been determined. Hypotheses include (i) gene silencing by the influence of telomere position; (ii) uniparental disomy of chromosome 20; (iii) deleted genes in the chromosomal segment, or (iv) effect of ring instability in cellular functions [26]. In this study, we investigated genotype-phenotype correlations through deleted genes in 20q13.33 using systems biology approaches to explain the associated clinical spectrum in our patient. With this approach, we identified candidate genes that may be involved in the pathophysiology of ring chromosome 20. To measure the importance of the protein-protein interaction network of genes located in the deleted region (Figure 1B), we examined the topological properties of the network using centrality analyses.

The proteins ARFGAP1, HELZ2, and EEF1A2 presented a high node degree in the network. These nodes are considered hubs and have central functions in a biological network [25]. ARFGAP1 serves as a regulator of vesicular trafficking of proteins [27]. HELZ2 is a helicase that acts as a transcriptional coactivator [28]. EEF1A2 promotes the GTP-dependent binding of aminoacyl-tRNA during protein biosynthesis and plays a role in the regulation of actin function and cytoskeletal structure. *EEF1A2* knockout mice showed degeneration of neurons in the spinal cord and brain stem [29], and heterozygous mutations in the gene were associated with intellectual disability, developmental delay, autistic behaviors, and epilepsy [30, 31]. Therefore, the EEF1A2 protein may be a good candidate to explain some of the symptoms present in individuals with deletion 20q13.33.

CHRNA4, ARFRP1, GID8, and SRMS were the nodes identified as bottlenecks in the network. ARFRP1 plays a role in membrane trafficking between the trans-Golgi network and endosomes. GID8 is a nuclear retention factor for *β*-catenin during Wnt signaling, and SRMS nonreceptor-type tyrosine kinases are a BRK family of kinases (BFKs) involved in the proliferation or differentiation of keratinocytes [32–34]. *CHRNA4* encodes alpha-4 nicotinic acetylcholine receptor subunits, and different mutations in the gene cause autosomal dominant nocturnal frontal lobe epilepsy (ADNFLE) [35, 36]. *CHRNA4* is a known gene associated with epilepsy in ring 20 patients [26]. Bottlenecks are related to the control of information between the interactions in the network [25, 37]; therefore, the identification of CHRNA4 already linked to the syndrome phenotype indicates that the haploinsufficiency of these bottlenecks could play a role in the development of our patient's phenotype.

Hub-bottlenecks (H-B) are nodes with a high degree and betweenness score. Among the nodes classified as H-B are two proteins, PTK6, another member of BFK, interacts directly with SRMS in the network. PTK6 functions as an intracellular signal transducer in epithelial tissues, contributing to the migration, adhesion, and progression of the cell cycle [38, 39]. COL9A3 is a structural component of hyaline cartilage, and mutations in the gene are associated with multiple epiphyseal dysplasia [40, 41].

Genes associated with the same illness have been observed to interact with each other more frequently than predicted by chance [42]. Therefore, we performed cluster analysis to examine densely connected regions in the final network and observe these novel candidate genes localized in 20q13.33 [43]. We analyzed a total of 11 clusters (data not shown). Interesting relationships were found; for example, the proteins HELZ2, EEF1A2, DIDO1, YTHDF1, PTK6, COL9A3, and COL20A1 interact with one another in cluster 2 (Figure 2C). Many of these genes are deleted in nonmosaic ring chromosome 20, and the clinical abnormalities identified in these individuals include findings also seen in our patient as seizures, intellectual disability, and developmental delay [8]. Ring chromosome 20 is associated with refractory epilepsy, behavioral problems, and mild-to-severe cognitive impairment. *De novo* microdeletion of 20q13.33 is associated with intellectual disabilities, developmental delay, speech and language delay, seizure, and behavioral problems such as autistic spectrum disorder. However, there is no pattern of abnormalities that would arouse clinical suspicion of a r(20) or *de novo* 20q13.33 microdeletion [19].

**Figure 3:**
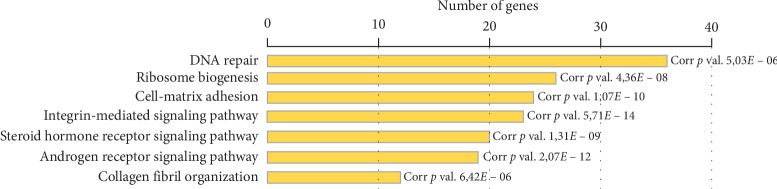
Summary of the bioprocess enrichment identified in cluster 2. The colored horizontal bars show GO terms frequently present.

Functional enrichment analysis in the clusters revealed that the candidate genes were enriched in several biological processes, including microtubule-based movement, nucleosome assembly, DNA repair, and cholinergic synaptic transmission (Tables [Supplementary-material supplementary-material-1]–[Supplementary-material supplementary-material-1]). Defects in these bioprocesses are associated with the development of neurological diseases, intellectual disability, neuropathies, and seizures [44–48]. In addition, such bioprocesses as cell-matrix adhesion and integrin-mediated signaling were identified (Figure 3). These pathways are involved in neural stem cell differentiation, neuronal migration, neuroplasticity, maturation, and function of synapses in the peripheral and central nervous system and may have an important contribution to the emergence of intellectual disability and seizures in humans [49–51]. The biological processes involving candidate genes denote the heterogeneity of pathways disrupted by 20q13.33 deletions.

The ring chromosome associated with subtelomeric deletions and duplications can confound and limit genotype-phenotype correlations. In fact, the circular structure of a ring chromosome, as described in this report, can change the ordinary 3D conformation of the chromatin in various ways and thus alter the expression of the genes present in the ring chromosome. The presence of an amplification identified in our ring chromosome analysis hinders efforts to determine its impact on the patient's phenotype. The duplication can be seen as a consequence of the mechanism of ring formation; in this case, an inverted duplication may be stabilized not only through telomere healing and telomere capture but also through circularization in the chromosomal ring [12]. Recently, chromothripsis and chromoanasynthesis have been proposed as two independent mechanisms that could explain the combination of deletions and duplications on the same chromosome. Indeed, various molecular approaches, including whole genome sequencing, have shown that the concomitance of amplification, deletion, and ring chromosomes can be the result of a more complex rearrangement with respect to a ring chromosome with only the loss of extremities [52, 53]. In this case, it is expected that the final phenotype of the proband is not only the result of the abnormal dosage of deleted genes but also of the altered expression of duplicate genes present in two copies.

## Supplementary Materials

Supplementary Materials Table 1: list of GO terms identified by BiNGO in Cluster 1. Table 2: list of GO terms identified by BiNGO in Cluster 2. Table 3: list of GO terms identified by BiNGO in Cluster 3. Table 4: list of GO terms identified by BiNGO in Cluster 4. Table 5: list of GO terms identified by BiNGO in Cluster 5. Table 6: list of GO terms identified by BiNGO in Cluster 6. Table 7: list of GO terms identified by BiNGO in Cluster 7. Table 8: list of GO terms identified by BiNGO in Cluster 8. Table 9: list of GO terms identified by BiNGO in Cluster 9. Table 10: list of GO terms identified by BiNGO in Cluster 10. Table 11: list of GO terms identified by BiNGO in Cluster 11. Table 12: list of genes located in the 0.9 Mb smallest overlap region encompassing band 20q13.33.
